# Safety and tolerability of long-term apomorphine infusion in advanced Parkinson's disease: an Indian multi-center (APO-IND) experience

**DOI:** 10.1038/s41598-023-46003-4

**Published:** 2023-10-31

**Authors:** Vinod Metta, Rajinder K. Dhamija, Lucia Batzu, Rukmini Mrudula, Natuva Sai Sampath Kumar, Arunan S., Cristian Falup-Pecurariu, Carmen Rodriguez-Blazquez, Vinay Goyal, Prashanth L.K., Kalyan Bhattacharya, Suresh Kumar, Kallol Ray Chaudhuri, Rupam Borgohain

**Affiliations:** 1 Department of Neurosciences, Institute of Psychiatry, Psychology and Neuroscience and Parkinson’s Foundation Centre of Excellence, King’s College London, King’s College Hospital, London, UK; 2 Parkinson’s Foundation Centre of Excellence, King’s College Hospital, London, Dubai, United Arab Emirates; 3 Parkinson’s Foundation Centre of Excellence, King’s College Hospital London, London, SE5 9RS UK; 4 Institute of Human Behavior and Allied Sciences, Lady Hardinge Medical College and SSK Hospital, New Delhi, India; 5 Institute of Movement Disorders, Narayana Medical College and Postgraduate Research Centre, Nellore, India; 6 Nizams Institute of Medical Sciences, Hyderabad, Telangana India; 7 SRM Institute of Medical Sciences and Technology, Chennai, India; 8 Department of Neurology, Transilvania University of Brasov, Brasov, Romania; 9 Carlos 111, Health Institute, Madrid, Spain; 10 Institute of Movement Disorders and Parkinson’s Centre, Medanta Hospital, New Delhi, India; 11 Center for Parkinson’s Disease and Movement Disorders, Manipal Hospital, Bangalore, India; 12 R G Kar Medical College and Hospital, Kolkata, India; 13 Amrita Institute of Medical Sciences, Kochi, India

**Keywords:** Neuroscience, Neurology

## Abstract

Advanced Parkinson’s Disease (APD) is complicated by the emergence of motor and non-motor fluctuations, which are initially predictable and eventually become unpredictable, in part due to erratic gastric absorption and short half of oral levodopa. Attempts to manage such fluctuations with oral dopaminergic drugs often lead to disabling dyskinesias. Continuous Subcutaneous Apomorphine Infusion (CSAI), despite being approved for the treatment of APD since 1993, was approved in India only in 2019. We studied the safety, tolerability and efficacy of CSAI in Indian patients with APD in a registry design to raise local awareness of this important treatment. We conducted a prospective registry-based observational audit at 10 centers across different states of India. Patients with APD, not responding to or with significant side effects from oral dopaminergic therapy, were assessed at baseline and at month 6 and 12 following CSAI infusion. Fifty-one patients completed the study, CSAI significantly reduced the functional impact of dyskinesia (p < 0.01 at 6 months and p < 0.001 at 12 months). There was a significant improvement in the OFF-state from baseline (p < 0.01 at 6 months and p < 0.001 at 12 months) No discernible side effects were observed apart from mild site reaction (n = 7), nausea (n = 7) skin nodules (n = 2). CSAI demonstrated safety, efficacy, tolerability and improved quality of life in patients with APD, as shown in previous studies. Our study highlighted current existing inequalities in treatment availability, lack of awareness, knowledge gap, affordability and cost remains a concern regarding apomorphine use in Indian PD population.

## Introduction

Parkinson’s disease (PD) is the second most common neurodegenerative disease affecting two in every 100 people over 60 years of age and as of 2022, more than 10 million people worldwide are living with PD^1^. As per 2016 estimate, over 0.58 million people are living with PD in India and a substantial rise in prevalence has been anticipated^2,3^. Unlike most neurodegenerative disorders, PD has effective symptomatic treatments that improve the quality of life (QoL) of patients to a great extent. Many of the clinical symptoms in early PD can be effectively alleviated by efforts to augment dopaminergic activity with levodopa, relevant enzyme inhibitors, and dopamine agonists. As patients age, disease progression causes erratic gastric emptying, poor levodopa absorption, increase and frequent dosing of pulsatile oral levodopa regimen, often leading to a variety of motor fluctuations, together with other limitations^4,5^. Apomorphine is the oldest non-ergot dopamine agonist that has consistently shown symptomatic benefit in PD with an efficacy similar to that of levodopa, though with a shorter onset and duration of action^6^. Apomorphine administered as continuous or intermittent subcutaneous infusion, is safe and effective in management of levodopa-related motor complications^7^. It is increasingly being recommended in Europe^8^ for people with motor fluctuations and advanced PD after being approved for the treatment of motor fluctuations in 1993. In India, however, CSAI was approved for usage in patients with APD only in 2019^9^ and is still not approved for use in the USA. The delay illustrates the growing concern about inequalities in healthcare and medication availability in different areas of the world, where need for availability of advanced therapies for PD, such as in India is paramount. Our efforts at creating an Indian registry of apomorphine use in PD patients is directed towards raising awareness of this important therapy among neurologists, patient groups and industry who need to ensure that therapies such as apomorphine infusion need to be made available to bespoke populations where a large number of PD patients would be diagnosed.

We investigated the safety, tolerability and efficacy of apomorphine administered as continuous subcutaneous infusion in patients with PD in 10 centers across India.

## Methods

This prospective, observational, multicenter study was conducted between 2021 and 2023 in 10 centers across India. Data was collected from the registries of the following centers: the Sri Ramaswamy Memorial (SRM) Institute for Medical Sciences, Chennai; Radha Gobinda Kar (RG Kar) Medical College and Hospital, Kolkata; Lady Hardinge Medical College, Delhi; Institute of Movement disorders and Parkinson’s research centre Medanta, hospital Gurugram; Manipal Hospital, Bengaluru; Narayana Medical College, Nellore; Amrita Institute of Medical Science and Research Centre, Kochi; Nizam’s Institute of Medical Sciences, Hyderabad; Institute of Movement Disorders Parkinson’s Centre of Excellence, City Neurosciences, Hyderabad; and RM institute of Neurosciences, Kochi. The study was conducted with collaboration and oversight by the Parkinson Foundation Centre of Excellence at the King’s College Hospital, London and the Transylvania University of Brasov, Brasov, Romania. The statistical analysis of the data was carried out at the Carlos III Health Institute, Madrid, Spain.

Procedural aspects of apomorphine administration and titration as per the UK NICE (The National Institute for Health and Care Excellence, England) guidelines^10^ and the protocol adopted at the Parkinson’s Centre of Excellence at King’s College Hospital, London under guidance and supervision of Professor Ray Chaudhuri, Dr Vinod Metta and Rupam Borgohain. Carmen Rodriguez Blazquez (CRB) acted as an independent data analyzer. Cristia Falup-Pecurariu (CFP) was the head of oversight on data and procedures. Data capture was in line with PD non-motor longitudinal study protocol as is in progress in London since 2011^11^. The ethical approval was obtained from the ethics committee of Nizam Institute of Medical Sciences, Hyderabad **(**NRES 10/H/2021). All the participants signed a written informed consent. Strict confidentiality of patient information was maintained throughout the study. The study was executed according to the principles of International Council for Harmonization of Technical Requirements for Pharmaceuticals for Human Use-Good Clinical Practice (ICH- GCP) and the Declaration of Helsinki.

Hoehn and Yahr (H&Y) Scale was used to stage their functional disability^12^. The study included patients with advanced idiopathic PD (as per H&Y staging 2.5 and above) who did not respond to the conventional oral dopaminergic treatments or developed side effects. “Non-responders” was used as a descriptive term in this study. Patients who were unable to tolerate the high oral dose of levodopa needed to obtain optimal levodopa levels or who responded better to non-oral therapies such as subcutaneous apomorphine were referred to as “non-responders” in this study. Patients with severe cognitive impairment, psychiatric conditions, other syndromes that mimicked PD, atypical parkinsonian features or any previous history of severe adverse effects like hallucinations, dopamine dysregulation syndrome and impulse control disorder from oral dopaminergic agonists were appropriately excluded. patients were grouped into PD subtypes based on clinical exam determined by experts/neurologists/movement disorder specialists. The study relied on cumulative experience of movement disorders experts who led the included centers and oversight was provided by KRC (Parkinson’s Disease Centre of Excellence, London) and CFP (Brasov Centre of Excellence, Romania).

The demographic parameters of the study subjects were noted at baseline. Each subject received 14 waking hours of CSAI from 8 am to 10 pm at the rate of 2 mg/h optimized to 3.0 mg/h at 6 months and 3.5 mg/h at 12 months. Titration was done by the treating PD specialist as driven by individual patients’ clinical responsiveness. Levodopa equivalent daily dose (LEDD) was calculated to estimate the overall baseline intake of total oral dopaminergic medications^13^.

Motor and non-motor assessments were performed using the Movement Disorder Society unified Parkinson’s disease rating scale (MDS-UPDRS) part 2, 3, 4, the non-motor symptom scale (NMSS), and the 8-item Parkinson's disease questionnaire (PDQ-8) on quality of life^14–16^. Anxiety and depression were evaluated using the hospital anxiety and depression scale (HADS), cognition was evaluated using Montreal cognitive assessment (MoCA) scale and the severity of fatigue was assessed using Parkinson’s disease fatigue scale (PFS-16)^17–19^. Parkinson’s disease sleep scale (PDSS) was used to quantify the level of sleep disturbances^20^. The King's Parkinson’s disease pain scale (KPPS) was used to quantify and assess the different types of pain perceived by the study subjects^21^. The outcomes were measured at baseline, at 6-month and 12-month follow-up visits. Any adverse drug reactions (ADRs) were documented on the suspected ADR reporting form authorized by the Pharmacovigilance Programme of India (PvPI). The patients’ data collected on the case record forms were analyzed using SPSS v20.0. Numerical data were expressed in terms of mean, standard deviation (SD) and range. Categorical data was expressed in terms of frequency (n) and percentage (%). Chi-Square tests, Wilcoxon test, etc. were used as mentioned in table legends, ANOVA was applied to evaluate the results of the study and any difference was considered statistically significant at p < 0.05.

### Ethical approval

This prospective, observational, multicenter study was conducted between 2021 and 2023 in 10 centers across India. Ethical approval was obtained from the respective institutional or independent ethics committees. All participants signed a written informed consent. Strict confidentiality of patient information was maintained throughout the study. The study was executed according to the principles of International Council for Harmonization of Technical Requirements for Pharmaceuticals for Human Use-Good Clinical Practice (ICH-GCP) and the Declaration of Helsinki.

### Informed consent

Informed consent was obtained from the patients, caregivers, and all participants involved in this study.

## Results

**Figure 1:**
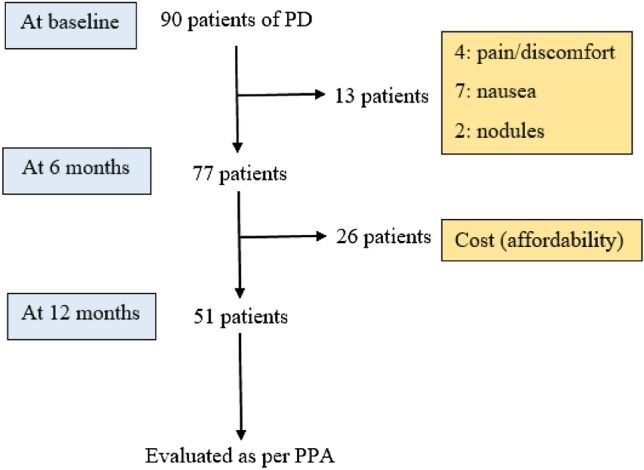
Trial profile. *PPA* per protocol analysis.

After screening, 90 patients with PD who satisfied the inclusion and exclusion criteria were recruited from 10 centers across different states/cities (Hyderabad, Chennai, New Delhi, Kerala, Andhra Pradesh, Kolkata, Rajasthan, Bengaluru) over a period of 18 months. At the 6-month follow up, 13 subjects (14%) dropped out of the study due to adverse effects (Fig. 1). Subsequently, another 26 patients (out of the remaining 77) discontinued the study due to cost and affordability issues. Hence, a total of 39 out of 90 subjects withdrew from the study at the 12-month follow up visit, raising the total dropout rate to 43%. Fifty-one patients completed the study (Fig. 1), hence a per protocol analysis was performed for these patients.

The mean age of the participants was 64.59 ± 7.12 years (range 45–80 years, Table 1). There was a male preponderance (62.7% males). The average duration of PD was 7.43 ± 2.03 years.

**Table 1:** Baseline characteristics of the patients (N = 51).

Age in years	
Mean ± SD	64.59 ± 7.12
Range	45–80
Sex [n (%)]	
Male	32 (62.7%)
Female	19 (37.3%)
Type of PD [n (%)]	
Akinetic dominant	27 (52.9%)
Tremor dominant	6 (11.8%)
Mixed	18 (35.3%)
Mean duration of PD (± SD) in years	7.43 ± 2.03
Range of duration of PD in years	4–14

*SD* standard deviation, *PD* Parkinson’s disease.

At baseline, most patients had H&Y scores (Table 2) of 3.0 or more. At 6 months, there was a variable change. However, with sustained use, by month 12, all analyzed patients had H&Y scores of 2.5 or less. More than one-third of the patients had H&Y score of 2.0.

**Table 2:** Hoehn and Yahr staging of Parkinson’s disease at baseline and following subcutaneous infusion of apomorphine.

HY stage*	Baseline n (%)	Month 6 n (%)	p^a^	Month 12 n (%)	p^b^
2.0	–	–	< 0.001	19 (37.3)	0.013
2.5	2 (3.9)	3 (5.9)		32 (62.7)	
3.0	34 (66.7)	25 (49.0)		–	
3.5	13 (25.5)	21 (41.2)		–	
4	2 (3.9)	2 (3.9)		–	

^a^Chi-square test for baseline vs month 6 scores; ^b^Chi-square test for baseline vs month 12 scores; *Hoehn and Yahr stage of Parkinson’s disease. p < 0.05 was considered significant.

LEDD scores significantly decreased from baseline at month 6 (p < 0.001) as CSAI was initiated and optimized and there was a significant improvement in UPDRS parts 3 and 4, NMSS total score, PDQ-8 summary index, depression and anxiety subdomains of HADS, KPPS and PFS-16 scores (p < 0.001). The same parameters showed sustained significant improvement (p < 0.001) at 12 months (Table 3). Following initiation of CSAI, all the clinical parameters showed a significant improvement from baseline at month 6 and month 12 (both p < 0.05), except in MoCA and PDSS (Table 3).

**Table 3:** Changes following subcutaneous infusion of apomorphine in patients with Parkinson’s disease (n = 51).

Time period	Baseline	Month 6	p^a^	Month 12	p^b^	p^c^
Measure	Mean ± SD (range)	Mean ± SD (range)		Mean ± SD (range)		
Dose of medication						
LEDD	823.73 ± 267.17 (400–1800)	574.51 ± 169.52 (200–1000)	< 0.001	407.84 ± 132.43 (200–800)	< 0.001	< 0.001
Apomorphine	–	24.67 ± 4.19 (18–36)	–	36.00 ± 6.79 (24–60)	–	< 0.001
UPDRS						
Part 2^d^	30.18 ± 1.85 (27–33)	23.41 ± 2.30 (19–27)	< 0.001	–		
Part 3	46.78 ± 4.21 (39–57)	34.96 ± 2.03 (30–39)	< 0.001	34.43 ± 0.86 (33–36)	< 0.001	n.s.
Part 4	17.78 ± 2.33 (13–22)	14.02 ± 1.42 (12–18)	< 0.001	11.98 ± 1.52 (9–15)	< 0.001	< 0.001
NMSS total^e^	128.06 ± 18.33 (87–161)	95.51 ± 100.0 (68–118)	< 0.001	78.00 ± 21.32 (46–124)	< 0.001	< 0.001
PDQ-8 Summary index	59.56 ± 7.45 (43.75–75.0)	42.50 ± 5.54 (37.5–53.13)	< 0.001	43.75 ± 5.56 (37.5–53.13)	< 0.001	n.s.
HADS-Depression	12.22 ± 2.06 (8–18)	10.51 ± 1.43 (6–14)	< 0.001	10.51 ± 1.43 (6–14)	< 0.001	n.s.
HADS-Anxiety	13.02 ± 7.75 (9–18)	10.31 ± 1.53 (6–14)	< 0.001	10.51 ± 1.43 (6–14)	< 0.001	n.s.
MOCA	24.55 ± 2.77 (20–30)	24.55 ± 2.77 (20–30)	n.s.	24.59 ± 2.66 (20–30)	n.s.	n.s.
PFS-16	11.16 ± 3.66 (0–16)	9.33 ± 2.78 (0–12)	< 0.001	9.82 ± 3.03 (0–16)	< 0.001	n.s.
PDSS	63.55 ± 18.07 (20–90)	66.18 ± 12.63 (30–90)	n.s.	66.18 ± 12.36 (30–90)	n.s.	n.s.
KPPS	40.59 ± 8.56 (21–67)	33.69 ± 9.10 (20–60)	< 0.001	33.29 ± 9.23 (20–60)	< 0.001	n.s.

^a^Wilcoxon test for baseline vs month 6 scores; ^b^Wilcoxon test for baseline vs month 12 scores; ^c^Wilcoxon test for month 6 vs month 12 scores; ^d^*SD* Standard deviation, *LEDD* Levodopa equivalent daily dose, *UPDRS* Unified Parkinson’s disease rating scale, *NMSS* non motor symptoms scale, *PDQ-8* 8-item Parkinson's disease questionnaire, *HADS* hospital anxiety and depression scale, *MOCA* Montreal cognitive assessment, *PFS-16* Parkinson’s disease fatigue scale, *PDSS* Parkinson’s disease sleep scale, *KPPS* King’s PD Pain Scale.

When compared to baseline, at 6 months, significant improvement was seen in UPDRS parts 2, 3, and 4, total NMSS score, PDQ-8 summary index, HADS-Depression, HADS-Anxiety, PFS-16, and KPSS scores. From 6 to 12 months, significant improvement was noticed in UPDRS parts 3 and 4, total NMSS score, PDQ-8 summary index, HADS-Depression, HADS-Anxiety, PFS-16, and KPSS. As compared to baseline, at 12 months, significant improvement was seen in only in UPDRS part 4 and total NMSS score. Other parameters also showed improvement though it was not statistically significant. Only two pain parameters, i.e., nocturnal pain and orofacial pain, showed significant improvement at month 6 and month 12 from baseline following subcutaneous apomorphine administration (p < 0.05, Table 4).

**Table 4:** Changes in KPPS domains in patients with Parkinson’s disease following subcutaneous infusion of apomorphine.

Timeline	Baseline	Month 6	p^a^	Month 12	p^b^	p^c^
Measure	Mean ± SD (range)	Mean ± SD (range)		Mean ± SD (range)		
Musculoskeletal pain	2.65 ± 1.820 (0–6)	2.55 ± 1.689 (0–6)	n.s.	2.55 ± 1.689 (0–6)	n.s.	n.s.
Chronic pain	2.31 ± 1.581 (0–4)	2.55 ± 1.689 (0–6)	n.s.	2.55 ± 1.689 (0–6)	n.s.	n.s.
Fluctuation-related pain	2.51 ± 1.725 (0–6)	2.51 ± 1.725 (0–6)	n.s.	2.51 ± 1.725 (0–6)	n.s.	n.s.
Nocturnal pain	16.27 ± 4.574 (11–24)	11.06 ± 1.363 (9–14)	< 0.001	10.18 ± 2.463 (2–14)	< 0.001	n.s.
Oro-facial pain	11.16 ± 3.657 (0–16)	9.33 ± 2.783 (0–12)	< 0.001	9.82 ± 3.031 (0–16)	< 0.001	n.s.
Discoloration, oedema/swelling	2.84 ± 2.336 (0–10)	2.84 ± 2.336 (0–10)	n.s.	2.84 ± 2.336 (0–10)	n.s.	n.s.
Radicular pain	2.84 ± 2.336 (0–10)	2.84 ± 2.336 (0–10)	n.s.	2.84 ± 2.336 (0–10)	n.s.	n.s.

^a^Wilcoxon test for baseline vs month 6 scores; ^b^Wilcoxon test for baseline vs month 12 scores; ^c^Wilcoxon test for month 6 vs month 12 scores; p < 0.05 was considered significant; *SD* standard deviation.

No discernible side effects were observed apart from mild site reaction (n = 7), nausea (n = 7), and skin nodules (n = 2). No deaths were reported.

## Discussion

Apomorphine and its usage was first studied by Matthiessen and Wright^22^, who in 1868 synthetized apomorphine hydrochloride by heating morphine with concentrated hydrochloric acid. It was trialed at the end of nineteenth century and beginning of twentieth century and clinically used to treat a range of psychiatric conditions like mania, hysteria, schizophrenic elation and other alcohol related disorders^23^.

In these studies, spontaneous erection was noted as an unexpected effect, which would much later lead to the commercialization of apomorphine as an agent to treat erectile dysfunction^24^. However, its use has been limited due to its emetic properties and its poor bioavailability when administered orally. In 1884, it was De Weil and colleagues^7^, who first hypothesized that apomorphine could be useful in patients with PD and, almost 70 years later, Schwab and colleagues^25^ demonstrated marked improvement in rigidity and tremor.

This opened the door for successful introduction of subcutaneous injections and continuous infusion of apomorphine by Stibe et al.^26^ in 1980, demonstrating remarkable efficacy in reducing the “OFF” periods in patients with advanced PD. Today, apomorphine is known as a dopamine agonist for the treatment of advanced PD. Apart from a few anecdotal audits and one single-centric study by Deva Kumar et al.^27^ with a short-term follow up of one month, which showed considerable improvement in the ON periods with reduction in motor fluctuations and manageable side effects, no other studies, to our knowledge, investigated the safety, tolerability and efficacy of CSAI in the Indian population using a registry based observational record of real-life clinical practice. Our study is the first multi-centric (across 10 different states/cities) study conducted in India on CSAI and has long-term (12 months) follow-up data based on the apomorphine registry created in conjunction with the advanced therapies international center at Kings college hospital, London.

In our study, the effects of CSAI on motor fluctuations were remarkably consistent. Subcutaneous apomorphine was effective in significantly decreasing OFF period fluctuations as indicated by UPDRS part 4 (Table 2), improving dyskinesias and correspondingly, reducing oral levodopa doses as reported by earlier apomorphine infusion studies^28^. We noticed a significant reduction in LEDD following CSAI initiation (p < 0.001 at both 6 months and 12 months from baseline). CSAI also led to a significant improvement in the functional impact of dyskinesia (p < 0.01 at 6 months and p < 0.001 at 12 months) and time spent in dyskinesia (p < 0.001 at both 6 months and 12 months). Our findings are consistent with those of the TOLEDO study^29^ which also revealed a significant increase in the amount of time without bothersome dyskinesia in the apomorphine arm (p = 0.0008), and also as shown in previous studies^30,31^. Our findings are also in line with other studies demonstrating a significant reduction in the severity of dyskinesia up to 65% with subcutaneous apomorphine^32,33^. However, some studies with subcutaneous apomorphine reported no significant change in dyskinesia severity^34^. This discrepancy may be because of the delivery trajectories used with apomorphine, concomitant levodopa optimization as well as use of rescue pulsatile strategies. Subcutaneous apomorphine fills in the gaps in motor function in people who experience frequent or unexpected OFF periods because of its non-oral route and ability to provide continuous drug delivery^34^. In our study “painful OFF state dystonia” showed significant improvement from baseline (p < 0.01 at 6 months and p < 0.001 at 12 months). However, the “time spent in OFF” did not show any significant difference. Non motor symptoms in PD are widely reported to be under-rated, under-treated, under-diagnosed^35^. The NMSS (Non Motor Symptom Scale) is a comprehensive instrument that offers a thorough grading (of both severity and frequency) of 30 distinct non-motor symptoms in PD across nine different domains^16^. Our study showed that all the NMSS domains, except memory, improved significantly at both 6- and 12-months post CSAI. Our study showed that CSAI also improved both upper gastrointestinal (GI) symptoms like dysphagia, drooling of saliva and lower GI symptoms as measured by the relevant gastrointestinal domain of NMSS as reported by many previous studies^36,37^. Pain is one of the important non-motor symptom of PD that can occur at any stage with an estimated prevalence of 40–83%^38^ and can be a predictor of quality of life^39^. A few categories of pain identified with PD include musculoskeletal pain, PD-related chronic pain, fluctuation-related pain, nocturnal pain, orofacial pain and peripheral limb/abdominal pain^40,41^. Only some of these subtypes, such as dystonia-related musculoskeletal pain, painful restless leg syndrome respond well to dopaminergic treatment^42^. In our study, orofacial and nocturnal pain domains showed substantial improvement post CSAI. No other pain domains on the KPPS scale showed any beneficial improvement. This finding was in line with Haddad et al.^43^ suggesting that apomorphine has no impact on pain processing (pain threshold and pain-induced cerebral activity), further urging additional studies to explore the possible function of apomorphine in sensory symptoms like pain etc.

One striking feature of our study was the cost factor and affordability. In Indian patients, these aspects play a crucial role for continuity of the therapy. In our cohort, in fact, 29% of patients discontinued CSAI because of affordability issues and only 14.4% withdrew due to adverse effects [nausea (7.8%), local pain/discomfort (4.4%) and skin nodules at the infusion site (2.2%)]. This is analogous to earlier studies^44^ where side effects with CSAI like nausea, vomiting, subcutaneous nodules and bruising at the site of injection have been reported^45^. Nausea, an expected dopaminergic side effect of apomorphine, usually manifests at the initiation of the drug administration^46^. However, it is mild and can be taken care of with preventive anti-emetics such as domperidone^47^. There is a need for randomized controlled trials with a larger study sample that can shed more light on the effectiveness of subcutaneous apomorphine over the conventional ones like subthalamic nucleus Deep Brain Stimulation (STN-DBS) or levodopa–carbidopa intestinal gel (LCIG). Personalizing treatment choices requires evidence and clinical experience based guidance for the device-aided therapies in PD^4,48^. In two other prospective, multicenter, international, real-life cohort observation studies^49,50^ comparing all three device-aided treatments (CSAI, LCIG, and STN-DBS), all three treatment options had a beneficial effect on depression and anxiety. Aspects of sleep dysfunction (insomnia, excessive daytime sleepiness, and restless leg syndrome) and fatigue improved with both LCIG and bilateral STN-DBS compared with CSAI^49,50^, whereas CSAI showed a beneficial effect on perceptual problems and hallucinations^49,50^. All three (STN-DBS, CSAI, and LCIG) had beneficial effects on the miscellaneous domain of the NMS scale, which incorporates unexplained pain, olfaction, weight changes, etc.^49,50^.

Ours is first long-term follow-up Indian registry-based study to raise awareness among all health care professionals involved in PD care. The data can be extrapolated to other developing countries too. In line with other several previous studies, our data showed beneficial effects over functional disability with many returning to Hoehn and Yahr stage 2–2.5 (from H&Y 3.5) at 12 months and had positive effects on the total NMSS score and overall QoL of PD patients. Affordability and availability, major factors for continuity of therapy in Indian patients, are the challenges that need to be overcome (many had to discontinue apomorphine because of the cost) and one that industry needs to take increasing note of by making apomorphine available at affordable costs in developing or low-income countries. Our study showed that CSAI has a beneficial effect on both motor and non-motor symptoms of PD and the fact that it can be used as an option in those who are not candidates for surgical therapies, such as deep brain stimulation, extends its range of utilization. The recently published APOKADO study shows that home initiation of CSAI is feasible and improves patients’ QoL more quickly with the same level of tolerance as with in-hospital initiation^51^. This approach is less expensive and may make it easier for patients to access CSAI.

## Conclusion

CSAI is safe, effective, with good tolerability in Indian patients with APD. Patient selection, participation, education and dose optimization enhance improved outcomes and perhaps, implementing cost effectiveness strategies can influence sustainability and continuity of these therapies.

## Data Availability

Raw data were generated at the King’s College Hospital, London, Dubai. The data supporting the findings of this study are available from the corresponding author, VM, upon request.
